# Gut Microbiome and Its Associations With Acute and Chronic Gastrointestinal Toxicities in Cancer Patients With Pelvic Radiation Therapy: A Systematic Review

**DOI:** 10.3389/fonc.2021.745262

**Published:** 2021-12-06

**Authors:** Jinbing Bai, Zahra A. Barandouzi, Claire Rowcliffe, Rebecca Meador, Despina Tsementzi, Deborah Watkins Bruner

**Affiliations:** ^1^ Nell Hodgson Woodruff School of Nursing, Emory University, Atlanta, GA, United States; ^2^ Winship Cancer Institute, Emory University, Atlanta, GA, United States; ^3^ Rollins School of Public Health, Emory University, Atlanta, GA, United States

**Keywords:** cancer, gut microbiome, gastrointestinal side effects, treatment toxicity, pelvic radiation therapy

## Abstract

**Aim:**

Pelvic radiation therapy (RT) can impact the gut microbiome in patients with cancer and result in gastrointestinal (GI) toxicities. The purpose of this systematic review was to describe the effects of RT on the gut microbiome and the associations between the gut microbiome and GI toxicities in patients treated with pelvic RT.

**Methods:**

PubMed, Embase, and Web of Science databases were searched from their earliest records to August 2020. The articles screening process adhered to the Preferred Reporting Items for Systematic Reviews and Meta-Analyses guidelines. The Mixed Method Assessment Tool was used to assess the methodological quality for each included study. All study findings were synthesized and presented in narrative format. Thirteen studies were included. The gut microbiome of fecal samples was analyzed using 16S rRNA sequencing approaches.

**Results:**

There were disparities in alpha and beta diversities that existed across the studies. Divergent results were found among various phyla, including *Firmicutes, Bacteroidetes, Proteobacteria, Actinobacteria, Cyanobacteria*, *Fusobacteria*, and *Deinococcus-Thermus*. Moreover, alteration in the gut microbiome diversity and abundance related to cancer treatment was associated with pelvic toxicities, specifically diarrhea. Following treatment, increases in the abundance of *Bacteroides* was associated with diarrhea and radiation enteritis.

**Conclusions:**

Pelvic RT can disrupt the diversity and abundance of commensal gut microorganisms. A dysbiotic gut microbiome showed a promising association with radiation enteritis through alterations of the intestinal barrier function, innate immunity, and intestinal repair mechanisms; however, confounders, such as diet, were not thoroughly addressed.

## Introduction

Cancer is a major public health problem across the world, and it is the second leading cause of death in the United States (1, 2). In 2020, a total of 1,806,590 new cancer cases and 606,520 cancer deaths were estimated to occur in the United States (2). Pelvic cancers, such as gynecological cancer, prostate cancer, and colorectal cancer, are among the most frequently diagnosed cancers (1, 2). Pelvic radiation therapy (RT) is a core modality primarily used to treat pelvic cancers (3, 4), with the purpose of reducing tumor size to allow surgical resection of tumors or killing tumor cells that cannot be removed *via* surgeries (5). With the advancement of cancer treatment modalities, including pelvic RT, the cancer mortality rate has continuously decreased, with an estimated 16.9 million cancer survivors currently alive in the United States (2, 6, 7). However, the wide use of RT has resulted in a high incidence of radiation‐induced symptoms and toxicities (8, 9). Specifically, 90% of cancer patients with pelvic RT develop a permanent change in their bowel habits (4, 10), and they may develop pelvic radiation toxicities such as radiation enteritis, radiation proctitis, and radiation cystitis post treatment (4, 11). As these patients with pelvic cancers live longer, an increasing number of them suffer for decades with RT-related gastrointestinal (GI) symptoms and toxicities, all of which can significantly decrease the patients’ quality of life (QOL).

Current knowledge has delineated the microbiome-host interactions as an integrative point in the pathogenesis of GI symptoms and toxicities among cancer patients receiving RT (12, 13). The human microbiotas and their genomes in the GI tract are collectively called the gut microbiome (14), which varies among different hosts and across different parts of the GI tract within a single host (15, 16). The gut microbiome is a dynamic ecosystem and is highly susceptible to a variety of environmental factors (e.g., diet and physical activity) and host-driven factors (e.g., cancer diagnosis and tolerance of RT) (17, 18). The pathogenesis of the gut microbiome for cancer treatment-related symptoms and GI toxicities may be related to the following pathways: inflammatory cytokines, intestinal permeability, bacteria translocation; changes in the epithelial surface microbiota pattern, intestinal protection from noxious stimuli, epithelial repair mechanisms; and the release of immune cells and molecules (19). Thus, pelvic RT can lead to a dysbiotic gut microbiome, potentially increasing intestinal permeability, thereby causing GI symptoms and toxicities.

Recently, research has started to investigate the interaction of the gut microbiome with pelvic RT and its associations with RT-related GI toxicities. Both animal models and clinical studies have corroborated decreases in diversity and disturbance of the gut microbial communities across cancer treatments (12, 13). Based on male C57/Bl6 mice, Johnson et al. found that radiation led to significant decreases in anaerobic *Enterobacteriaceae* and *Lactobacillus* genera (20); Kim and colleagues found a significant increase in *Alistipes* and a decrease in *Mucispirillum* genera when characterizing the mouse gut microbiome during radiation (21). Among human cancer populations, gynecological cancer patients with pelvic RT showed a higher abundance of *Actinobacteria* but a lower abundance of *Fusobacteria* compared to healthy individuals (22). Patients with pelvic tumors who experienced acute post-RT diarrhea showed a decrease in microbial diversity compared to both healthy volunteers and patients who received pelvic RT but without diarrhea; patients with diarrhea exhibited an increased abundance of *Actinobacteria* and *Bacilli* but a decreased abundance of *Clostridia* compared to patients without diarrhea (23). All these studies seem to indicate that patients receiving RT exhibit marked changes in their gut microbiome, and these changes may be associated with GI symptoms and toxicities, such as diarrhea and radiation enteritis.

Although evidence has shown the potential role of the gut microbiome in GI symptoms and toxicities during cancer treatments, current literature has primarily focused on the impact of chemotherapy and immunotherapy on the gut microbiome (12, 13). Some animal studies have proposed the potential role of the gut microbiome as a protection against RT toxicities, suggesting a higher abundance of *Lachnospiraceae* and *Enterococcaceae* associated with less RT side effects in mice models (24). Although some evidence has shown the potential associations of the gut microbiome with symptoms and GI toxicities in cancer patients, findings of studies are inconsistent, and the role of pelvic RT on the gut microbiome and resulted symptoms remain obscure (12, 13).

Radiation enteritis and symptoms experienced by cancer patients treated with RT is a global challenge. Understanding the impact of pelvic RT on the gut microbiome and its associations with symptoms and GI toxicities may provide a precise target to relieve symptoms and decrease patients’ suffering. The current literature reviews primarily focused on preclinical and clinical studies to elucidate the underlying mechanisms of gut microbiome dysbiosis in relation to RT (8, 25). Also, to date, no systematic review was conducted with a robust methodology to evaluate the quality of the included studies using a valid tool (25). Thus, a systematic review was needed to synthesize current research regarding the impact of pelvic RT on the gut microbiome and its relationship with symptoms and GI toxicities in adult cancer patients with pelvic cancers.

## Aims

A systematic review was conducted to examine: 1) the influence of pelvic RT on the gut microbiome in adult patients with pelvic cancers receiving RT; and 2) the associations between the gut microbiome and clinician-reported and patient-reported GI toxicities and symptoms among adult patients treated with pelvic RT.

## Methodology

### Search Strategy

With the guidance of the Preferred Reporting Items for Systematic Reviews and Meta-Analyses (PRISMA) framework (26), a systematic review was conducted by searching three databases: PubMed, Embase, and Web of Science. Key terms used to search these databases included GI microbiome, gut microbiome, gut microflora, intestinal bacterial, microbiota composition, radiation, radiation injury, radiation toxicities, radiotherapy, pelvic radiotherapy, pelvic irradiation, and brachytherapy. All three databases were searched from their earliest records to August 2020. Detailed information for the searching process is displayed in supplementary **Table S1**. The literature search process was overseen by a health science librarian from Emory University in Atlanta, Georgia, United States.

### Study Selection

All the articles identified from the database searches were initially screened *via* title and abstract by one author (CR) and subsequently confirmed by another author (ZB) from this study. Then, all full texts for the remaining articles were retrieved and independently reviewed by two reviewers (CR and ZB) to determine study eligibility. Ambiguous studies were discussed and reviewed by the independent reviewers (CR and ZB) together with a third author (JB). Eligibility criteria for articles to be included were as follows: 1) studied the gut microbiome with next generation sequencing (e.g., 16S rRNA gene amplicon) in patients receiving pelvic RT; 2) focused on pelvic cancer populations; and 3) published in English. Articles were excluded from this study if they: 1) studied an animal model or other non-human models; 2) did not focus on pelvic cancer; 3) did not study the gut microbiome or gut microbiota with next generation sequencing approaches; or 4) were supplementary information, patents, abstracts without available full texts, or unpublished reports. When multiple studies with similar results were found to be authored by the same research team, only the most recent publication was included in this review. A secondary search of reference lists was conducted to decrease the likelihood of omitting eligible articles from this review.

### Data Extraction

A standard data extraction form was developed by our team to extract data from eligible studies. Information that was extracted from each study included: 1) characteristics of the studies according to the Johns Hopkins evidence level and quality guide (27) (e.g., authors, year of publication, origin of the study, research purpose, study design, and sample size); 2) participant information (e.g., demographics, clinical information, inclusion and exclusion criteria); 3) variables, measures, and data collection strategies; 4) data analysis; and 5) research findings and related interpretations. All study data were extracted by two authors (CR and ZB), and one author (JB) confirmed the accuracy of data extraction.

### Assessment of Study Quality

The Mixed Methods Assessment Tool (MMAT) is a scoring system designed to evaluate complex systematic literature reviews, including qualitative studies, quantitative studies (e.g., randomized controlled trials [RCTs], quasi-experimental studies, and descriptive studies), and mixed methods studies (28). Studies eligible for this review represent many types of research, so the MMAT was appropriate for quality assessment. The MMAT was updated in 2018, and the updated version of this tool was utilized for this review. This tool includes two screening criteria (applicable to all types of studies) and five domain criteria (specific to each study design), resulting in seven criteria for each study. All these questions have three response options (i.e., “yes”, “no”, or “can’t tell”) (28). An overall quality score ranges between 20% (one criterion met) and 100% (all criteria met), which is calculated using the number of domain criteria met divided by five. This tool has moderate to high reliability and has been widely used for the critical evaluation of more than 50 systematic reviews (29). The quality assessment of the included studies attempted to provide a comprehensive overview of their value and to address any potential weaknesses.

### Data Synthesis and Analysis

Descriptive statistics were used to describe all the eligible studies. All study findings were synthesized and presented in tables and figures using a narrative format.

## Results

### Literature Search

**Figure 1:**
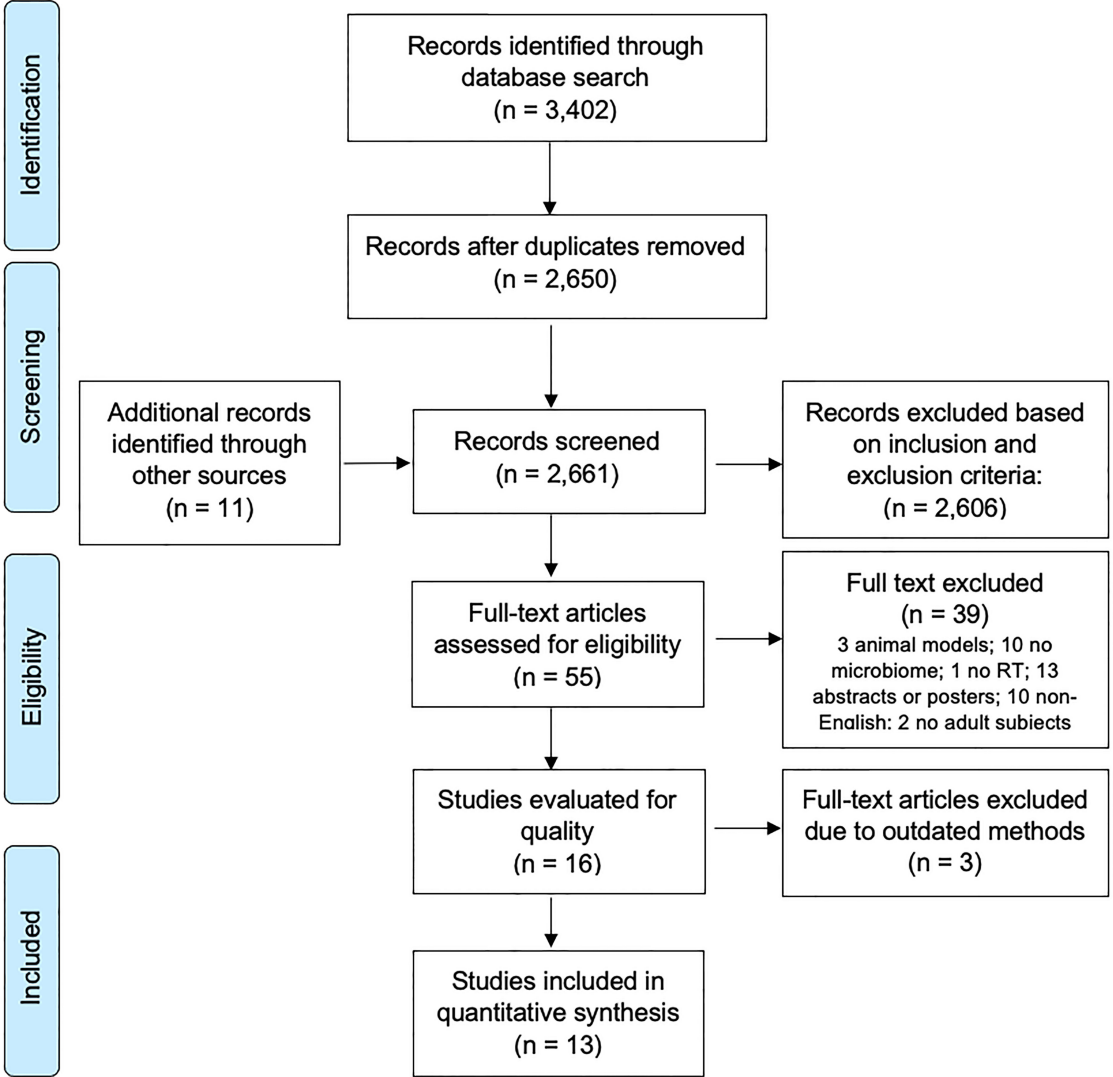
PRISMA flow diagram of the studies of the gut microbiome and RT-related gastrointestinal toxicities.

We initially identified 3,402 reports for possible inclusion in this systematic review by searching three databases: PubMed (n = 92), Web of Science (n = 1905), and Embase (n = 1405). Eleven additional records were identified *via* the reference articles of related review papers. After reviewing the articles’ titles and abstracts, duplicate articles and other irrelevant reports (n = 3358) were removed, resulting in 55 reports requiring full text review. Among these 55 full texts, we excluded studies of animal models (n = 3), reports not including the gut microbiome (n = 10) or RT (n = 1), conference abstracts or poster presentations (n = 13), full articles in a language other than English (n = 10), and articles in which the study population did not include adults (n = 2), leading to 16 studies left for study quality review. Three articles were further excluded due to outdated methods in the gut microbiome sequencing. Thus, a total of 13 studies were included in this systematic review (**Figure 1**).

### Study Characteristics and Sampling

Among these 13 included studies, 11 of them were quantitative descriptive studies, one was a quantitative non-randomized study, and one was a RCT. The sample sizes of the included studies varied from 5 to 134 participants. These studies were conducted between 2008 and 2020, primarily in the United States (n = 4), China (n = 3), South Korea (n = 2), Malaysia (n = 1), Finland (n = 1), France (n=1) and the United Kingdom (n = 1). All the included studies collected stool samples. In addition, two of the studies also collected blood samples, and one study collected gut biopsies. The gut microbiome data were sequenced using 16S rRNA targeting various gene regions. Radiation-induced GI clinician reported toxicities or patient reported symptoms were reported by seven studies, with most studies primarily focusing on diarrhea. **Table 1** describes characteristics and sampling of these included studies.

**Table 1:** Characteristics of the Included Studies (n = 13).

Authors, Year, Country	Study design	Sample size, Subjects, Mean age or age range	Biospecimen	Microbiome pipeline	Symptoms/Toxicities	Tools/Measures
Manichanh, Varela, et al 2008, France (23)	Cross-sectional, quantitative-descriptive	10 patients with endometrial, rectum and uterus cancers and 5 healthy controls	Stool	16S rRNA position 968–1401 in *E. coli*	Diarrhea	Common Terminology Criteria
Nam, Kim, et al 2013, South Korea (22)	Prospective observational study, quantitative-descriptive	9 patients with stage I-IIB gynecologic cancer; age range: 35-63 years	Stool	454 sequencing of 16S rRNA V1-V2 region	NA	NA
Wang, Ling, et al 2015, China (30)	Cross-sectional, quantitative-descriptive	11 patients with cervical, anal, and colorectal cancer; age range: 41-64 years	Stool	454 sequencing of 16S rRNA V3 region	Fatigue, diarrhea	Common Terminology Criteria for adverse Events (CTCAE), Multidimensional Fatigue Inventory
Sze, Baxter, et al 2017, US (31)	Cross-sectional, quantitative-descriptive	67 patients with colorectal cancer, Mean age: in patients with adenoma: 61.68 years; in advanced adenoma: 63.11 years; and in carcinoma: 61.65 years	Stool	Illumina MiSeq of 16S rRNA V4 region	NA	NA
Youssef. Lahti, et al 2018, Finland (32)	Cross-sectional, quantitative-descriptive	96 patients with stomach, pancreas, small intestine, colon, and rectal cancer; age range: 19-98 years	Stool	Ion-Torrent PGM of 16S rRNA V2, V3, V4, V8, V6-7, and V9 regions	NA	NA
Ferreira, Andreyev, et al 2019, UK (33)	Observational study with three cohorts. Prospective cohort (early cohort), cross sectional (late cohort), nested case/control (colonoscopy cohort)	134 patients with prostate cancer (32 in the early cohort, 87 in the late cohort, and 15 in the colonoscopy cohort [cases = 9, controls = 6]); age range: in early cohort 63-72 years, in late cohort 68-79 years; in colonoscopy cases 68-79 years and colonoscopy controls 57-69 years	Stool, gut biopsy, and blood	Illumina MiSeq of 16S rRNA V1-V2 region	Diarrhea, procitis, sphincter control, tenesmus, belleding and pain	Clinician- and patient-reported outcomes
Wang, Wang, et al, 2019, China (34)	Prospective cohort, quantitative-descriptive	18 patients with stage II-IV cervical cancer; age range: 30-67 years	Stool and peripheral blood	Illumina HiSeq of 16S rRNA V4 region	Grade 1-3 radiation toxicity including pain, tenesmus, rectal bleeding, fecal incontinence, diarrhea and vomiting	Clinical symptoms and medical history
Ding, Li, et al 2020, China (35)	Quantitative-nonrandomized	5 patients with endometrial and cervical cancer; age range: 45-81years	Stool	Illumina MiSeq of 16S rRNA V4-V5 region	Diarrhea, rectal hemorrhage, abdominal/rectal pain, fecal incontinence, functional status, and cirrhosis	Radiation Therapy Oncology Group, CTCAE, Kamofsky Performance Status
Gonzalez-Mercado, Henderson, et al 2021, US (36)	Cross-sectional, quantitative-descriptive	31 patients with stage II-III rectal cancer; mean age: 60.8 years	Stool	Illumina MiSeq of 16S rRNA V3-V4 region	Fatigue, sleep disturbance, and depression	Patient-Reported Outcome Measures Information System-Fatigue, Reported Outcome Measures Information SystemSleep Disturbance, Hamilton Depression Rating Scale
Gonzalez-Mercado, Lim, et al 2020, US (36)	Cross-sectional, quantitative-descriptive	56 patients with rectal cancer; mean age: 60.5 years	Stool	llumina MiSeq of 16S rRNA V3-V4 region	NA	NA
Jang, Chang, et al 2020, South Korea (37)	Cross-sectional, quantitative-descriptive	45 patients with rectal cancer; mean age: 57 years	Stool	Ion-Torrent PGM of 16S rRNA V1-V2 region	NA	NA
Mitra, Biegert, et al 2020, US (38)	Cross-sectional, quantitative-descriptive	35 patients with stage IB1-IVA cervical cancer; age range: 35-72 years	Stool	Illumina MiSeq of 16S rRNA V4 region	Bowel and urinary toxicity	Expanded Prostate Cancer Index (EPIC)
Rosli & Shahar, et al 2020, Malaysia (39)	Double-blind randomized controlled trial	30 patients with endometrial, cervical, prostate, colon, and rectal cancer (intervention = 14 control = 16); mean age in intervention and control groups: 57 years 55 years	Fecal microbiome transplant	qPCR quantification of Bifidobacterium with specific primers	Diarrhea	Patient-Generated Subjective Global Assessment

CTCAE, Common Terminology Criteria for adverse Events; EPIC, Expanded Prostate Cancer Index; NA, not applicable; NI, no information; SD, standard deviation; UK, United Kingdom; US, United States.

### Study Quality Assessment

Based on the MMAT quality scoring system, most of the included studies had an overall quality score of 80% or above: 80% (n = 6) and 100% (n = 5). Two studies had a quality score of 60% due to the small sample size and potential risk of inappropriate measures. Of the studies with an overall quality score of 80%, the criterion that was not met was primarily associated with the sampling strategy relevant to address the research question. Based on this assessment, none of the eligible studies were excluded from this review. **Supplementary Tables S2**, **S3** display the quality scores of these quantitative non-randomized studies (n = 12) and RCTs (n = 1), respectively.

### Impact of Pelvic RT on the Gut Microbiome

A total of 11 studies were reviewed to show the impact of pelvic RT on changes in gut microbiome diversity (alpha diversity and/or beta diversity) and taxa abundance of the gut microbiome (**Table 2**). These studies focused on a variety of pelvic cancers, including gynecological cancers only (n = 3), colorectal cancers only (n = 4), prostate cancer only (n = 1), and mixed pelvic cancers, such as gynecological, colon, and prostate cancers (n = 3). Most of the included studies reported the gut microbiome composition pre- to post-RT or chemoradiation therapy (CRT) (30–32, 34, 36, 37, 40); two studies evaluated the gut microbiome changes pre-RT, during RT, and post-RT (22, 33); two studies assessed the gut microbiome pre-RT and during RT (38, 39); and one study assessed the composition of the gut microbiome only post-CRT (37).

**Table 2:** Effects of Radiation Therapy on the Gut Microbiome in Patients with Pelvic Cancers.

Authors, year	Participants	Comparisons	Gut Microbiome Diversity and Composition
Nam, Kim, et al 2013 (22)	9 gynecologic cancer patients treated with RT or CRT	Patients at four time points from pre- to post-treatment, (T0 = baseline *vs* T1 = first RT, T0 *vs* T2 = end of the fifth RT, T0 *vs* T3 = post-RT)	Changes at T1 compared to T0: ↑Streptococcaceae at T1; ↓Weissella confuse, Enterobacter sp. mcp11b, Klebsiella pneumonia, and Adlercreutzia equolifaciens at T1; Changes at T2 compared to T0: ↓ α‐diversity (estimated OTUs) at T2; ↑Fusobacteria, Fusobacteriaceae, Butyrate-producing bacterium SS2/1, Human intestinal firmicute CB47, Clostridiales bacterium DJF CP67 at T2; ↓Eubacteriaceae, Ruminococcuscallidus, Dialistersp. E2 20, Eubacterium hallii, Actinomycesodontolyticus, and Lactobacillus murinus at T2; Changes at T3 compared to T0: ↓ α‐diversity (unique OTUs) at T3; ↑Veillonellaceae, Enterococcaceae, Lactobacillales bacterium, Butyrateproducing bacterium, Ruminococcus sp.DJF VR52, Prevotella copri, Ruminococcus sp. CO28, Butyrate-producing bacterium T1-815, Roseburia inulinivorans, Bacteroides sp. CCUG 39913, Swine fecal bacterium FPC110, Faecalibacterium sp. DJF VR20, Clostridium methylpentosum, Oscillospira sp. BA04013493, Candidatus Bacilloplasma, Clostridales bacterium A2-162, Coriobacterium sp. CCUG 33018, Amphibacillus sp. YIM-kkny6, Lachnospiraceace bacterium DJF RP14, Clostridium leptum, Ruminococcus sp. CS1 at T3; ↓Firmicutes, Eubacteriaceae, Prevotella stercorea, Clostridium sp. BG-C36, at T3
Wang, Ling, et al 2015 (30)	11 cervical, colorectal, and anal cancers with RT	Patients with diarrhea pre- *vs* post-RT and patients without diarrhea pre- *vs* post-RT	↓ α‐diversity (i.e., Chao1 and Shannon) in patients with and without diarrhea post-RT; ↓ ratio of *Firmicutes* to *Bacteroidetes* from 1.79 to 0.83 in patients without diarrhea and from 2.15 to 0.63 in patients with diarrhea at post-RT; ↑*Bacteroides*, ↑*Clostridium XIVa*, ↓*Faecalibacterium*, ↓*Lachnospiracea*, ↓*Oscillibacter*, ↓*Roseburia*, and ↓*Streptococcus* at genus level in patients with and without diarrhea post-RT
Sze, Baxter, et al 2017 (31)	67 colorectal cancer patients (adenoma, advanced adenoma, and carcinoma) treated with surgery, chemotherapy, and RT	Patients pre- *vs* post-treatment (for three groups: carcinoma, adenoma, and advanced adenoma); patients *vs* healthy controls post-treatment	Significant difference in beta diversity pre- to post-treatment in carcinoma patients, but no difference in adenoma and advanced adenoma; Comparing three categories of patients with healthy people after treatment showed shared microbiome profile among patient groups (e.g., *Faecalibacterium*, *Lachnospiraceae*, *Bacteroides*, *Dorea*, *Anaerostipes*, and *Roseburia*); Post-treatment samples from patients with carcinoma more closely resemble those of a normal colon; this pattern was not observed for the other 2 groups; Patients with carcinoma showed higher similarity compared to healthy colon post-treatment, but there was a non-significant increase in similarity with healthy colon in adenoma and advanced adenoma post-treatment groups
Youssef, Lahti, et al 2018 (32)	96 stomach, pancreas, small intestine, colon, and rectal cancer patients before RT or chemotherapy or rectal, stomach, and small intestine after RT or chemotherapy	Treated patients *vs* non-treated patients	↑*Lactobacillaceae* in treated patients at family level; ↑*Lactobacillus* in treated patients at genus level
Ferreira, Andreyev, et al 2019 (33)	32 patients with prostate cancer followed for 12 months with and without RE: Clinician-reported outcome (CRO) gastrointestinal toxicity and patient-reported outcome (PRO) gastrointestinal toxicity	Patients with *vs* without radiation enteropathy (RE) at each time point pre to post-RT	↓ α‐diversity (Chao1) over time in early cohort; ↓ *Clostridium* IV proportions, ↓ *Roseburia* with PRO as well as CRO over time in early cohort;
Wang, Wang, et al 2019 (34)	10 cervical cancer patients with radiation enteritis treated with pelvic RT	Radiation enteritis patients pre- *vs* post-RT	↓Prevotella_9, Bacteroides, Coprococcus, Desulfovibri post-RT; ↑Citrobacter Serratia, Roseburia, Prevotella_2, Pseudomonas, Veillonella, Sutterella and Megamonas post-RT
Gonzalez-Mercado, Henderson, et al 2021 (36)	24 rectal cancer patients with co-occurring symptoms treated with CRT	Patients with co-occurring symptoms pre- *vs* post-CRT	↓ α‐diversity (i.e., Pielou eveness) post-CRT; ↓ *Coprococcus* and *Desulfovibrio* post-CRT
Gonzalez-Mercado, Lim, et al 2020 (36)	56 rectal cancer patients from HPR and NHW ethnicity treated with CRT	HPR *vs* NHW patients post-CRT	↑*Pasteurellaceae* in HPR and ↑*Eubacteriaceae* in NHW at family level; ↑*Muribaculaceae*, *Prevotella 2* and *7*, *Gemella*, *Bacillales Family XI, Catenibacterium, Sutterella*, and *Pasteurellales* in HPR and ↑*Turicibacter* in NHW at genus level
Jang, Chang, et al 2020 (37)	45 rectal cancer patients treated with CRT who had complete response and non-complete response	Complete response *vs* non-complete response post-CRT	Two groups had significantly different gut microbial beta diversity (Bray-Curtis index); ↑*Acholeplasma*, ↑*Oribacterium*, ↓*Bacteroides* in complete response *vs* non-complete response groups at the genus level; ↑*Oxyphotobacteria* in complete response compared to non-complete response *at the class level*; ↑*Corynebacteriales* and *Acholeplasmatales* at the order level in complete response compared to non-complete response; ↑*Corynebacteriaceae*, ↑*Deinococcaceae*, ↑*Clostridiaceae*, ↓*Bacteroidaceae* and ↓*Rikenellaceae* in complete response compared to non- complete response at family level; Complete response rate had a positive linear relationship with *Dialister pneumosintes* and a negative linear relationship with *Anaerostipes hadrus*, *Bacteroides dorei*, and *Alistipes senegalensisJC50.* There was a V-shaped association between complete response rate and *Bacteroides spp*, *Marseille-P2653* and *Duode-nibacillus massiliensis.* The highest complete response rate (mean 98.7%) was associated with presence of *Duodenibacillus massiliensis*, *Dialister pneumosintes, and Bacteroides* sp. *Marseille*-*P2653* and absence of *Anaerostipes hadrus*, *Bacteroides dorei*, and *Alistipes senegalensis JC50*
Mitra, Biegert, et al 2020 (38)	35 cervical cancer patients with RT	Patients followed from baseline and weeks 1, 3, and 5 of treatment (T1, T2, T3, T4)	↓ alpha diversity (Shannon) over course of RT; ↓ alpha diversity (Shannon) in week 5 compared to baseline; Difference in beta diversity (Jaccard distance) over course of RT; ↓ *Clostridiales* in order level over course of RT; ↑*Phascolarctobacterium, Lachnospiraceae, Veillonella, Erysipelotrichaceae*, and *Fecalitalea* in patients with high toxicity at T4 compared to T1
Rosli, Shahar, et al 2020 (39)	23 endometrial, cervical, colon, rectal, and prostate cancer patients with RT or CRT	Intervention and control groups followed from day 0 to day 45 of RT (day 0 without RT and intervention, day 7 with intervention and without RT, days 14 and 28 with intervention and RT, and day 45 without intervention and with RT)	↑*Bifidobacterium* spp. after a week of PHGG consumption (day 7) which was doubled at day 14 at the initiation of RT in the intervention group; ↓*Bifidobacterium* spp. during the RT in intervention group; ↑*Bifidobacterium* spp. after a week of PHGG consumption (day 7) which was doubled at day 14 at the initiation of RT in the control group; ↓*Bifidobacterium* spp. during the RT in control group;

RT, radiation therapy; PHGG, partially hydrolyzed guar gum; CRT, chemoradiation therapy; FMT, fecal microbiome transplantation; HRP, Hispanic Puerto Ricans; NHW, Non-Hispanic Whites.

Dynamic changes of the gut microbial diversity were inconsistent with respect to pelvic RT. Some studies reported lower alpha diversity and different beta diversity during and at the end of RT (22, 38). One study showed significantly lower alpha diversity over time, up to one year after RT (33). Furthermore, other studies found lower alpha diversity and differences in beta diversity post-RT or post-CRT (30, 31, 37, 40). In contrast, some studies did not find significant changes in alpha and beta diversity post-RT or post-CRT (31, 32, 36, 37).

Significant gut microbial taxa were found to be associated with gut microbiome changes during and following pelvic RT compared to pre-RT. At the phylum level, a lower ratio of *Firmicutes* to *Bacteroidetes*, a decrease of *Firmicutes*, and an increase of *Fusobacteria* were reported during and post-RT compared to pre-RT (22, 30). At the order level, a decrease in the abundance of *Clostridiales* was observed over the course of RT (38). At the family level, an increase in the abundance of *Fusobacteriaceae* and *Streptococcaceae* and a decrease in the abundance of *Eubacteriaceae* were reported during and post-RT (22). At the genus level, a decrease in *Bacteroides, Prevotella_9, Coprococcus, Desulfovibri, Faecalibacterium, Lachnospiracea, Oscillibacter, Roseburia*, and *Streptococcus* and an increase in the abundance of *Sutterella, Prevotella_2, Serratia, Pseudomonas, Veillonella*, *Megamonas, Bacteroides*, and *Clostridium_XIVa* were observed pre- to post-RT (30, 34). In contrast, Sze et al. observed post-treatment samples more closely resembled those of a normal colon (31).

In addition, some studies reported various taxa by comparing different patient groups at post-RT/post-CRT. At the genus level, González-Mercado et al. found higher abundance of *Muribaculaceae, Prevotella*, *Gemella, Bacillales Family XI, Catenibacterium, Sutterella, Pasteurellales*, and *Pasteurellaceae* in Hispanic Puerto Ricans compared to non-Hispanic whites; however, non-Hispanic whites were enriched in *Turicibacter* and *Eubacteriaceae* compared to the Hispanic Puerto Ricans at the end of CRT (36). Jang et al. reported higher abundance of *Corynebacteriales* and *Acholeplasmatales* at the order level; of *Corynebacteriaceae, Deinococcaceae*, and *Clostridiaceae* at the family level; and of *Acholeplasma, Oribacterium* at the genus level in the complete response group compared to the non-complete response group (37). Also, Youssef et al. reported higher abundance of *Lactobacillaceae* at the family level and *Lactobacillus* at the genus level in patients treated with either chemotherapy and/or RT compared with the non-treated control group (32).

### Associations Between the Gut Microbiome and Symptoms/GI Toxicities

A total of seven studies were reviewed to show the associations between the gut microbiome and symptoms/GI toxicities among cancer patients with pelvic RT (**Table 3**). These studies primarily focused on GI-related symptoms including diarrhea (n = 5), radiation enteritis (n = 1), and GI and urinary toxicities (n = 1).

**Table 3:** Associations of Radiation Therapy and the Gut Microbiome with Pelvic Toxicities.

Authors, year	Pelvic Toxicities	Acute vs Late	Comparisons	Gut Microbiome Diversity and Composition
Manichanh, Varela, et al 2008 (23)	Diarrhea	Unknown	Patients with and without diarrhea	↓ α‐diversity (Shannon) in patients with diarrhea compared to patients without diarrhea pre-RT; ↑ *Firmicutes* and *Actinobacteria* in patients with diarrhea after RT
Wang, Ling, et al 2015 (30)	Diarrhea	Acute	Patients with diarrhea vs healthy controls; Patients with diarrhea vs patients without diarrhea post-RT	↓ α‐diversity (Shannon) in patients who later developed diarrhea compared to healthy people and patients without diarrhea pre-RT; ↓ α‐diversity (Shannon index) in patients who developed diarrhea compared to patients without diarrhea post-RT; ↑ *Bacteroides, Dialister, Veillonella*, and ↓ *Clostridium XI* and *XVIII*, *Faecalibacterium*, *Oscillibacter, Parabacteroides*, and *Prevotella* in patients with diarrhea compared to patients without diarrhea pre-RT; ↓ in Firmicutes to Bacteroidetes ratio from 2.15 to 0.63 and ↑ in unclassified bacteria (*Phylum:others*) in patients with diarrhea post-RT; ↑* Alistipes*, *Bacteroides*, *Clostridium_XI*, *Erysipelotrichaceae*, *Escherichia, Lachnospiracea*, *Megamonas*, and unclassified (Genus: others), and ↓ *Clostridium* XIVa and *Sutterella* in patients with diarrhea compared to patients without diarrhea post-RT at genus level
Ferreira, Andreyev, et al 2019 (33)	Radiation enteropathy patients: Clinician-reported outcome (CRO) gastrointestinal toxicity and patient-reported outcome (PRO) gastrointestinal toxicity	Acute and late	Patients in early cohort* with and without radiation enteropathy pre-RT Patients in early cohort with and without radiation enteropathy over time Patients in late cohort** with and without radiation enteropathy pre-RT	↑ α‐diversity (Chao1) in early cohort with no radiation enteropathy at pre-RT; ↑ *Clostridium* IV proportions with PRO at Pre-RT as well as CRO in early cohort; ↑ *Roseburia* proportions with PRO at Pre-RT as well as CRO in early cohort; ↑ *Phascolarctobacterium* proportions with CRO at Pre-RT as well as PRO in early cohort; ↑ *Roseburia* with CRO over time in late cohort ↑ SCFA-related microbial metabolic pathways with symptoms in early cohort; ↓ fatty acid metabolism pathways with rising CRO diarrhea grade in late cohort; *Roseburia* significantly rose with both actual and historical clinician-reported diarrhea grade 3 and grade 0-2; No significant difference at either phylum or genus levels in patients with PRO; However, *Roseburia* was significantly associated with toxicity, and diarrhea
Wang, Wang, et al 2019 (34)	RE: combination of clinical symptoms (e.g. abdominal pain, tenesmus, rectal bleeding, fecal incontinence, diarrhea or vomiting without other obstructive symptoms)	Unknown	Non-RE vs RE group RE1 (grade 1 radiation toxicity) vs RE2 (grade 2 radiation toxicity) vs RE3 (grade 3 radiation toxicity)	↓ α‐diversity (Simpson and Shannon) in RE group; The two groups had significantly different gut microbiomes in terms of β‐diversity (unweighted Unifrac) More heterogeneous gut communities among RE patients compared to non-RE ↑ Proteobacteria in RE group (37.1% of total bacterial community on average) compared to non-RE (15.9% on average); At the class level, ↑*Gammaproteobacteria* in RE group compared to non-RE; At the order level, ↑*Enterobacteriales* and *Oceanospirillales* in RE group; At the family level, ↑*Enterobacteriaceae*, *Phyllobacteriaceae* and *Beijerinckiaceae*, ↓ *Bacteroidaceae* and *Ruminococcaceae* in RE group; At the genus level,↑*Serratia*, *Bacteroides, Megamonas, Novosphingobium*, *Prevotella* and *Prevotella-9* and ↓ B*lautia*, *Plebeius* and *Ruminococcaceae UCG‐003* in RE patients compared to non-RE; ↓ α‐diversity (Shannon) in RE3 compared to RE1; ↑ *Virgibacillus, Alcanivorax*, *Phenybacterium* and ↓ *Coprococcus*, *Collinsella*, *rc4_4* in RE1 compared RE2
Ding, Li, et al 2020 (35)	Eight weeks after FMT, there was ≥1-grade reduction in RTOG/EORTC late toxicity grade, diarrhea, rectal hemorrhage, abdominal/rectal pain, fecal incontinence, functional status, and cirrhosis in patients who received FMT compared to pre-FMT	Late	Patients with FMT vs donors (unpaired comparisons) Patients that received FMT at pre vs post-FMT (paired comparisons)	↑ α‐diversity (Shannon) and OTUs post-FMT compared to pre-FMT; Similar β‐diversity (unweighted UniFrac distances), post-FMT compared to their donors; Eight weeks post-FMT; Case 1: ↑* Phascolarctobacterium* and *Lachnoclostridium*, *Veillonella, Romboutsia* and *Escherichia* compared to pre-FMT; ↓ *Clostridia*, and *Gammaproteobacteria* compared to pre-FMT; Case 2: ↑ *Erysipelotrichia, Coriobacteriia*, and genus *Blautia, Faecalitalea, Lachnoclostridium* compared to pre-FMT; ↓ *Bacilli, Negativicutes, Streptococcus, Bacteroidia, Clostridia* and *Bacteroides* compared to pre-FMT; Case 3: ↑ *Alistipes*, *Phascolarctobacterium, Streptococcus* and *Bacteroides* compared to pre-FMT; ↓ *Faecalibacterium, Bacteroidia*, *Clostridia* and *Bacill* compared to pre-FMT
Mitra, Biegert, et al 2020 (38)	GI and urinary toxicity, EPIC	Acute	Patients with low and high GI and urinary toxicity over course of RT (baseline and weeks 1, 3, and 5 of treatment (T1, T2, T3, and T4)) Patients with high toxicity at T4 compared to T1	↑ α‐diversity (Shannon) in high GI toxicity over time; -significantly dissimilar microbiome communities were observed between patients with high and low toxicity at week 5 after RT; ↑ *Phascolarctobacterium, Lachnospiraceae, Veillonella, Erysipelotrichaceae*, and *Fecalitalea* in patients with high toxicity at T4 compared to T1; ↑ *Clostridiales* and *Desulfovibrio* in patients with low toxicity and ↑ *Sutterella*, *Finegoldia*, and *Peptococcaceae (Clostridia)* in patients with high toxicity at T1; ↑ *Clostridiales* and *Desulfovibrio* in patients with low toxicity and ↑ *Sutterella*, *Finegoldia*, and *Peptococcaceae (Clostridia)* in patients with high toxicity at T4
Rosli, Shahar, et al 2020 (39)	Diarrhea	Acute	Intervention group (IG) and control group (CG) followed from day 0 to day 45 of RT	↑ diarrhea in the IG who received PHGG, upon initiation of RT; ↓ diarrhea in the IG who received PHGG, at day 45 (end of pelvic RT); ↑ diarrhea in the CG from baseline to day 45 after end of RT

RT, radiation therapy; CRT, chemotherapy and radiotherapy; FMT, fecal microbiota transplantation; PHGG, partially hydrolyzed guar gum; RE, radiation enteritis; RTOG, Radiation Therapy Oncology Group; EORTC, European Organization for Research and Treatment of Cancer. Gonzalez-Mercado et al. (2021) , Gonzalez-Mercado et al. (2020), Nam et al. (22), Sze et al. (31), Jang et al. (37) and Youssef et al. (32) did not report the association between the gut microbiome and pelvic toxicity.

Studies reported contradictory results on the gut microbiome diversity in patients who were treated with RT and developed GI toxicities. Lower alpha diversity and significant dissimilarities of beta diversity were observed in patients with diarrhea/radiation enteritis post-RT compared to healthy controls and/or patients without diarrhea/radiation enteritis before RT (23, 30, 34). However, one study found that patients with higher GI toxicity had higher alpha diversity over time. Also, significant dissimilar microbiome communities (i.e., beta-diversity) were found between patients with low and high toxicity at week 5 after RT (38).

Various gut microbial taxa were found to be associated with diarrhea, bowel and urinary toxicities, and radiation enteritis. At the phylum level, a higher abundance of *Firmicutes*, *Actinobacteria* and *Proteobacteria* was reported in patients with diarrhea/radiation enteritis after RT compared to patients without diarrhea/radiation enteritis (23, 34). At the family level, cancer patients who developed at least two out of three symptoms (fatigue, depression, and sleep disturbance) had lower *Ruminococcaceae* (40). At the genus level, the relative abundance of *Alistipes, Bacteroides, Clostridium_XI, Erysipelotrichaceae, Escherichia, Lachnospiracea, Roseburia, Megamonas, Clostridium IV*, and *Phascolarctobacterium* were significantly higher, whereas *Clostridium_XIVa* and *Sutterella* were lower in patients with diarrhea compared to patients without diarrhea during and after RT (30, 33). Regarding bowel and urinary toxicities, an increase in the abundance of *Phascolarctobacterium, Lachnospiraceae, Veillonella, Erysipelotrichaceae*, and *Fecalitalea* was found in patients with high toxicity after RT compared to before RT. Meanwhile, a high abundance of *Clostridiales* and *Desulfovibrio* in patients with low toxicity and a high abundance of *Sutterella, Finegoldia*, and *Peptococcaceae (Clostridia)* in patients with high toxicity was reported from pre-RT to post-RT (38). González-Mercado et al. showed that cancer patients who developed at least two out of three symptoms had a higher abundance of *Bacteroides, Blautia1, Oscillibacter, Lactobacillus*, and *Blautia2* after CRT (40). In terms of radiation enteritis, the abundances of the of *Bacteroidaceae* family, *Bacteroides genus*, and *Plebeius* species were higher in the non-radiation enteritis group compared to patients with radiation enteritis (34). Furthermore, patients with Grade 1 radiation enteritis had higher abundance of genera *Virgibacillus, Alcanivorax*, and *Phenybacterium*; and patients with Grade 2 radiation enteritis were enriched in *Coprococcus, Collinsella*, and *rc4_4* (34).

### Interventions on the Gut Microbiome and GI Toxicities Across RT

Two intervention studies were assessed in this review. Ding et al. evaluated the efficacy of fecal microbiome transplant (FMT) on the gut microbiome pattern and symptoms in 5 women with endometrial and cervical cancers. Findings revealed that FMT increased alpha diversity and changed the composition of the microbiome in every recipient (35). Also, FMT can lower the Radiation Therapy Oncology Group/European Organization for Research and Treatment of Cancer (RTOG/EORTC) grade and improve diarrhea, rectal hemorrhage, abdominal/rectal pain, fecal incontinence, and cirrhosis (35). Rosli et al. reported that consumption of 10 g partially hydrolyzed guar gum (PHGG) twice daily from 14 days before RT increased the relative abundance of *Bifidobacterium species* at the initiation of RT; however, its abundance decreased during RT (39). The impact of PHGG on GI toxicities is unclear (39).

## Discussion

Thirteen studies were systematically synthesized to describe the role pelvic RT plays in influencing the gut microbiome and its associations with symptoms and GI toxicities among patients with various pelvic cancers. These studies indicated that pelvic RT could lead to dysbiosis by altering the diversity and abundance of the gut microbiome. A lower alpha diversity, dissimilarity in beta diversity, and decreased relative abundance of the healthy-associated gut microbes seemed to be associated with GI toxicities such as diarrhea and radiation enteritis. However, these studies have very specific limitations, including small sample size, using different tools to measure toxicities, diversity of patient populations, combination of multiple pelvic cancers at different stages, RT dose, use of concurrent chemotherapy as another systematic treatment, the use of diverse DNA sequencing approaches (e.g., 16S rRNA V1-V9 gene regions), and using different methodologies to describe the gut microbiome diversity and composition (i.e., definition of operational taxonomic unit (OTU), and sequencing read processing), all of which further complicate the interpretation of inconsistent findings. Moreover, cofounding factors such as diet, body mass index (BMI), physical activity, changes in BMI pre- to post-treatment, and use of antibiotics, were not systematically measured in the included studies, which may affect the results.

RT is a core modality used to treat pelvic cancers (41). Pelvic RT can disrupt the gut microbiome and lead to marked changes (e.g., promoting dysbiosis) in the gut microbiome (8, 13). Pre-clinical studies (via animal models) found that RT causes significant changes in the diversity and abundance of the gut microbiome (21), including a significant decrease in *Enterobacteriaceae* and *Lactobacillus* groups (20). Current findings were inconsistent in human populations. This systematic review found a lower alpha diversity (e.g., Chao1 index, Shannon index, and observed OTUs) among cancer patients with pelvic RT (22, 30, 33, 38) and significant differences in beta diversity (e.g., Jaccard distance metric) between patients with and without pelvic RT (37, 38); however, these findings were inconsistent with other studies (31, 32, 37). These differences in results can be explained due to small sample size (with a range between 5 and 134), studying various different pelvic cancers (e.g., gynecologic cancers, colorectal cancers, prostate cancer, and other GI cancers), and the use of multiple treatment modalities (e.g., RT alone and CRT). Importantly, the use of various 16S rRNA gene regions (i.e., V1-V9) for the microbiome data sequencing may further explain the discrepancies in these results.

Pelvic RT can lead to a dysbiosis of gut microbiome taxa, with a larger average variation of the relative abundance during RT compared to before RT and after RT (22). Specifically, cancer patients with pelvic RT showed decreased *Firmicutes (the phylum level)* (22)*, Eubacteriaceae (the family level)* (22)*, Clostridiales (the order level)* (38)*, Faecalibacterium (the genus level)* (30), and increased *Fusobacteria (the phylum level)* (22)*, Fusobacteriaceae (the family level)* (22), *Streptococcaceae (the family level)* (22)*, Pseudomonas (the genus level)* (34)*, Veillonella (the genus level)* (34)*, Megamonas (the genus level)* (34), *Bacteroides (the genus level)* (30), *Clostridium_XIVa (the genus level)* (30), and *Lactobacillus (the genus level)* (32) across pelvic RT. These significant changes in the gut microbiome are associated with cytotoxic chemotherapy or RT (8). For example, pelvic RT could lead to a significant change in the health status of the GI tract, including chronic inflammation or abnormal function of the epithelial cells, which might directly affect the gut microbial community. Although previous study has concluded that pelvic RT leads to increases in *Bacteroides* and *Enterobacteriaceae*, and decreases in *Bifidobacterium, Faecalibacterium prausnitzii*, and *Clostridium cluster XIVa* (13), this study suggested a variety of gut microbial taxa are disrupted during pelvic RT, such as *Bifidobacterium, Lactobacillus*, and *Faecalibacterium prausnitzii*, all of which have been examined as probiotics to treat a dysbiotic gut microbiome (8, 42, 43). Due to the limitations of study methodology and study design for this review, these probiotic studies would benefit from a future review.

RT-induced diarrhea affects more than 80% of cancer patients with pelvic RT (44), and this can cause significant treatment delays or dose reductions and diminish patients’ QOL (45, 46). The gut microbiome can impact RT-associated GI toxicity and diarrhea through two primary mechanisms: microbial translocation and dysbiosis (8). Specifically, a dysbiotic gut microbiome can promote the pathogenesis of radiation-induced GI mucositis (inflammation) (13) *via* modulating the oxidative stress and inflammatory processes, intestinal permeability, mucus layer composition, epithelial repair and ability to resist harmful stimuli, and expression and release of immune effector molecules in the intestine (19). In this study, our findings showed a lower alpha diversity (e.g., Shannon index) of the gut microbiome to be associated with worse diarrhea and radiation enteritis before and after RT (30, 34), as well as a significant difference in beta diversity between patients with low and high GI toxicities after RT (38). Findings of our study further indicated that a higher abundance of *Bacteroides, Phascolarctobacterium*, and *Roseburia* was associated with higher GI toxicities while *Clostridium XI, XIVa* and *XVIII*, and *Faecalibacterium* were associated with lower GI toxicities (30, 33, 38)*. Phascolarctobacterium* and *Roseburia* are short chain fatty acid (SCFA) producers that promote GI homeostasis and whose depletion has been associated with irritable bowel disease; this seems to contradict the findings of our review. One potential hypothesis is that these bacteria are part of an intestinal mucosa–associated community, and increased competition by potentially pathogenic bacteria leads to increased shedding in the stools of patients at risk of RT-induced GI toxicities (33, 47). *Clostridia cluster groups* (P. 48, 49) and *Faecalibacterium genus (* 50 *)* are potential probiotics with leading roles in the maintenance of gut homeostasis. For example, *Clostridia cluster XVIII* was linked to promotion of regulatory T-cell expansion and protection from colitis and allergic diarrhea, and this group was significantly less abundant in patients who developed diarrhea (51). Similarly, *Faecalibacterium* genus includes a protective commensal bacteria, such as *Faecalibacterium prausnitzii* (50). Therefore, depletion of protective commensal bacteria during pelvic RT can promote much more severe GI toxicities among patients with pelvic cancers.

Pelvic RT for pelvic cancers has frequently been found to increase the risk of radiation enteritis, and this may lead to longer hospitalizations and interruptions to the treatment plans of cancer patients (52). Previous work has examined the impact of RT on the gut microbiome by assuming that changes in the gut microbiome may affect the developmental course of radiation enteropathy (20, 53). Particularly, a linkage between the gut microbiome and RT-induced enteritis has been reported in germ-free animals (54). It is known that inflammatory pathways such as the NF-kB signaling pathway could be activated in response to RT (55), and commensal bacteria may regulate inflammatory responses as well. For instance, *Bacteroides thetaiotaomicron* and *Bifidobacterium infantis* are able to suppress NF-kB activation, whereas gut microbes in the *Clostridium XIVa group* have been reported to attenuate inflammation of the gut epithelium (56, 57). With respect to these promising microbial relationships, strategies such as FMT (35), prebiotics (39), and probiotics (58–60), used for increasing bacterial diversity and promoting the protective commensal bacteria in patients at risk for radiation enteritis, should be tested to understand whether they modify the course of RT-induced enteropathy.

This study has several limitations. According to our results, it is not possible to causatively link any of those significantly identified taxa to the etiology of RT-induced enteropathy. The current study synthesized data related to both acute and late toxicities related to RT. These promising findings should be further examined in a large sample size by considering confounding factors, such as diet, and using standard methods to measure symptoms and gut microbiome profiles so that confirmed findings can be used to improve the clinical practice among patients with cancer receiving pelvic RT. Secondly, radiation enteropathy has multiple causes, which are likely to have differential contributions from the gut microbiome. So far, no objective markers of radiation enteropathy have been defined, and there is no option but to rely on abnormal outcomes, primarily measured by clinician-reported toxicities or patient-reported symptoms. Finally, this study systematically reviewed all the articles using next generation sequencing and published in English only, which may lead to some data selection biases.

## Conclusions

Pelvic RT can disrupt the diversity and abundance of commensal gut microorganisms. A dysbiotic gut microbiome showed promising associations with pelvic radiation enteropathy and toxicities through alterations of intestinal barrier function, innate immunity, and intestinal repair mechanisms. The conflicting reports on microbial diversity and abundance may be attributed to cofounding factors within the complicated relationship between the gut microbiome and RT enteropathy. This may limit our ability to clearly define the unique contribution of the gut microbiome in RT enteropathy, and this important relationship warrants further investigations. A better understanding of the role of the gut microbiome in RT-induced symptoms and GI toxicities may lead to new therapeutic approaches and the identification of predictive markers of RT-induced GI toxicities.

## Data Availability Statement

The original contributions presented in the study are included in the article/**Supplementary Material**. Further inquiries can be directed to the corresponding author.

## Author Contributions

JB: conception of the study, methodology, developing the search strategy, supervision, drafting and editing the article, and funding acquisition. ZB: conception of the study, methodology, screening and extracting data, developing the search strategy, drafting, and editing the article. CR: methodology, screening and extracting data, developing the search strategy, drafting and editing the article. RM: drafting and editing the article. DT: methodology, screening and extracting data, assessing the risk of bias, and completing data synthesis. DB: conception of the study, drafting and editing the article. All authors contributed to the article and approved the submitted version.

## Funding

This work was partially supported by the National Institutes of Health/National Institute of Nursing Research (1K99NR017897-01 and 4R00NR017897-03).

## Conflict of Interest

The authors declare that the research was conducted in the absence of any commercial or financial relationships that could be construed as a potential conflict of interest.

## Publisher’s Note

All claims expressed in this article are solely those of the authors and do not necessarily represent those of their affiliated organizations, or those of the publisher, the editors and the reviewers. Any product that may be evaluated in this article, or claim that may be made by its manufacturer, is not guaranteed or endorsed by the publisher.

## Supplementary Material

Supplementary Table 1 Search Strategies.

Supplementary Table 2 Study Quality Assessment Based on MMAT Methodological Quality Criteria Checklist for Quantitative Studies.

Supplementary Table 3 Study Quality Assessment Based on MMAT Methodological Quality Criteria Checklist for Randomized Control Trials.
