# Supplementary material for: Gut Microbiome and Its Associations With Acute and Chronic Gastrointestinal Toxicities in Cancer Patients With Pelvic Radiation Therapy: A Systematic Review
Source: Front Oncol. 2021 Dec 6;11:745262. doi: 10.3389/fonc.2021.745262 (PMC8685326; doi:10.3389/fonc.2021.745262)
Supplement: Supplementary Table 1 — Search Strategies. [file Table_1.docx]

**Supplementary Materials**

**Table S1.** Search Strategies

| Database | Search Term | Number of Results | Data Searched |
| --- | --- | --- | --- |
| PubMed | (radiation[tw] OR “radiation-induced” [tw] OR “radiation induced” [tw] OR "Radiation Injuries"[Mesh] OR radiotherapy[tw] OR "Radiotherapy"[Mesh] OR irradiated[tw] OR irradiation[tw] OR “radiation toxicities” [tw] OR “radiation toxicity” [tw] OR brachytherapy OR "Brachytherapy/adverse effects"[Mesh] OR “ionizing radiation” [tw] OR "Radiation, Ionizing/adverse effects"[Mesh] OR “pelvic radiotherapy” [tw] OR “pelvic radiation” [tw] OR “pelvic irradiation” [tw] “Off-target toxicity” [tw] OR "Gastrointestinal Microbiome/radiation effects"[Mesh] OR "Gastrointestinal Tract/radiation effects"[Mesh]) AND ("Gastrointestinal Microbiome"[Mesh] OR “gut microbiome” [tw] OR “Gut Microbiomes” [tw] OR “gut microbiotic composition” [tw] OR “Gut Microflora” [tw] OR “Gut Microbiota” [tw] OR “Gut Microbiotas” [tw] OR “intestinal microflora” [tw] OR "Intestinal Flora"[tw] OR “intestinal bacterial” [tw] OR microbiota[tw] OR “microbiota composition” [tw] OR “Gastrointestinal Microbiomes” [tw] OR “indigenous intestinal microflora” [tw] OR “enteric bacteria” [tw] OR “Gut microbial dysbiosis” [tw] OR "Dysbiosis"[Mesh]) | 92 | 8/17/2020 |
| Web of Science | TS=(radiation OR “radiation-induced” OR “radiation induced” OR "Radiation Injuries" OR radiotherapy OR irradiated OR irradiation OR “radiation toxicities” OR “radiation toxicity” OR brachytherapy OR “ionizing radiation” OR “pelvic radiotherapy” OR “pelvic radiation” OR “pelvic irradiation” OR “pelvic radiation disease”) TS=("Gastrointestinal Microbiome" OR “gut microbiome” OR “Gut Microbiomes” OR “gut microbiotic composition” OR “Gut Microflora” OR “Gut Microbiota” OR “intestinal microflora” OR "Intestinal Flora" OR “intestinal bacterial” OR microbiota OR “microbiota composition” OR “indigenous intestinal microflora” OR “enteric bacteria” OR Dysbiosis) | 1,905 | 8/17/2020 |
|  |  |  |  |
| Embase | ('radiation'/de OR radiation OR “radiation-induced” OR “radiation induced” OR "Radiation Injury" OR radiotherapy OR irradiated OR irradiation OR “radiation toxicities” OR 'radiation toxicity'/exp OR brachytherapy OR 'brachytherapy'/exp OR “ionizing radiation” OR “pelvic radiotherapy” OR “pelvic radiation” OR 'pelvic radiation'/exp OR “pelvic irradiation” OR 'pelvic radiation disease'/exp) AND ('intestine flora'/exp OR "Gastrointestinal Microbiome" OR “gut microbiome” OR “Gut Microbiomes” OR “gut microbiotic composition” OR “Gut Microflora” OR “Gut Microbiota” OR “intestinal microflora” OR "Intestinal Flora" OR “intestinal bacterial” OR microbiota OR “microbiota composition” OR “indigenous intestinal microflora” OR “enteric bacteria” OR Dysbiosis OR 'dysbiosis'/exp) | 1,405 including 1 retracted article | 8/17/2020 |

**Table S2.** Study Quality Assessment Based on MMAT Methodological Quality Criteria Checklist for Quantitative Studies

| Appraisal Criteria | Screening Question 1. *Are there clear research questions?* | Screening Question 2.  *Do the collected data allow to address the research questions?* | Question 1.  *Is the sampling strategy relevant to address the research question?* | Question 2. *Is the sample representative of the target population?* | Question 3. *Are the measurements appropriate?* | Question 4.  *Is the risk of nonresponse bias low?* | Question 5.  *Is the statistical analysis appropriate to answer the research question?* | Quality Score (%) |
| --- | --- | --- | --- | --- | --- | --- | --- | --- |
| Manichanh, Varela, et al 2008^1^ | Yes | Yes | No | No | Yes | Yes | Yes | 60 |
| Nam, Kim, et al 2013^1^ | Yes | Yes | No | Yes | Yes | Yes | Yes | 80 |
| Wang, Ling, et al 2015^1^ | Yes | Yes | No | Yes | Yes | Yes | Yes | 80 |
| Sze, Baxter et al 2017^1^ | Yes | Yes | Yes | Yes | Yes | No | Yes | 80 |
| Youssef, Lahti, et al 2018^1^ | Yes | Yes | Yes | Yes | Yes | Yes | Yes | 100 |
| Wang, Wang, et al 2019^1^ | Yes | Yes | No | Yes | Yes | Yes | Yes | 80 |
| Ferreira, Andreyev, et al 2019^1^ | Yes | Yes | Yes | Yes | Yes | No | Yes | 80 |
| Ding, Li, et al 2020^2^ | Yes | Yes | No | Yes | No | Yes | Yes | 60 |
| Gonzalez-Mercado, Henderson, et al 2020^1^ | Yes | Yes | Yes | Yes | Yes | Yes | Yes | 100 |
| Gonzalez-Mercado, Lim, et al 2020^1^ | Yes | Yes | Yes | Yes | Yes | Yes | Yes | 100 |
| Jang, Chang, et al 2020^1^ | Yes | Yes | Yes | Yes | Yes | Yes | Yes | 100 |
| Mitra, Biegert, et al 2020^1^ | Yes | Yes | Yes | Yes | Yes | No | Yes | 80 |

Abbreviation: MMAT= mixed methods appraisal tool; ^1^ quantitative descriptive study ^2^ quantitative non-randomized study

**Table S3.** Study Quality Assessment Based on MMAT Methodological Quality Criteria Checklist for Randomized Control Trials

| Appraisal Criteria | Screening Question 1. *Are there clear research questions?* | Screening Question 2.  *Do the collected data allow to address the research questions?* | Question 1. *Is randomization appropriately performed?* | Question 2. *Are the groups comparable at baseline?* | Question 3. *Are there complete outcome data?* | Question 4.  *Are outcome assessors blinded to the intervention provided?* | Question 5. *Did the participants adhere to the assigned intervention?* | Quality Score (%) |
| --- | --- | --- | --- | --- | --- | --- | --- | --- |
| Rosli, Shahar, et al 2020 | Yes | Yes | Yes | Yes | Yes | Yes | Yes | 100 |

Abbreviation: MMAT = mixed methods appraisal tool
